# Integrating medical imaging datasets with blockchain wallets: A case study on ARDS-COVID19 patients

**DOI:** 10.1371/journal.pone.0338897

**Published:** 2026-03-18

**Authors:** Marco Esperança, Tiago Galvão, Diogo Freitas, Joao C. Ferreira, Ana Cysneiros, Luís Bento

**Affiliations:** 1 Faculty of Logistics, Molde University College, Molde, Norway; 2 ISTAR, Center for Research of Technologies and Architecture, Instituto Universitario de Lisboa ISCTE-IUL, Lisbon, Portugal; 3 3 INESC INOV, Lisbon, Portugal; 4 CHRC, NOVA Medical School, Faculdade de Ciências Médicas, NMS, FCM, Universidade NOVA de Lisboa, Lisboa, Portugal; 5 Intensive Care Department, Hospital de São José, Unidade Local de Saúde São José, Lisboa, Portugal; Beijing Technology and Business University, CHINA

## Abstract

Medical imaging plays a critical role in diagnosing and managing acute conditions such as Acute Respiratory Distress Syndrome (ARDS), particularly in intensive care settings. However, radiological data are often siloed across Picture Archiving and Communication Systems (PACS), with limited interoperability, traceability, and patient control. This paper proposes and validates a blockchain-enabled architecture that integrates radiological imaging data into a patient-controlled digital wallet (BioWallet) ecosystem. The system combines verifiable credentials, decentralized identifiers (DIDs), and a FHIR-compliant backend to ensure secure, auditable, and standards-based access to DICOM images and associated metadata. The ledger stores only consent and audit hashes, while clinical data remain off-chain and correctable within FHIR/EHR systems, ensuring auditability without hindering rectification. A validation scenario replicating an ICU emergency was conducted using synthetic ARDS-COVID19 cases to assess latency, consent enforceability, and user experience. Results showed a 67% reduction in image access time compared to traditional systems, 100% success in blocking unauthorized access, and high clinician satisfaction. The architecture supports FAIR-compliant reuse of annotated imaging datasets, enhances transparency in image-driven research, and aligns with GDPR and future European digital identity frameworks. This work demonstrates the feasibility and value of a decentralized, patient-centric approach to imaging data governance in high-stakes clinical environments.

## 1. Introduction

The effective and ethical reuse of clinical data remains one of the most pressing challenges in healthcare innovation. While medical images are central to diagnosis and clinical decision-making, their secure sharing across institutions is hampered by regulatory constraints, lack of interoperability, and non-standard annotation practices. This is particularly critical in high-acuity environments such as Intensive Care Units (ICUs), where real-time access to imaging and clinical data can directly impact patient outcomes. The COVID-19 pandemic, especially in the management of Acute Respiratory Distress Syndrome (ARDS) patients, exposed significant weaknesses in current systems—including fragmented records, manual consent workflows, and unstructured data formats—that hinder timely intervention, monitoring, and research scalability [1–3].

A major barrier to clinical data reuse, particularly for artificial intelligence (AI) development and federated research, lies in the absence of structured and standardized annotations accompanying medical images [4]. Although the DICOM format encapsulates metadata, IDs, and even structured reports, the latter are rarely used in practice. In radiology, most annotations are unstructured and embedded in free-text PDF reports, making querying and data retrieval nearly impossible [5]. Moreover, ICU imaging studies—such as chest X-rays—often lack any formal report, requiring correlation with other sources like clinical diaries or lab values scattered across different hospital systems [6]. This complicates the creation of high-quality datasets for training and validation of machine learning models, especially when temporal or pathological alignment is required.

In this context, two technological enablers offer promising solutions. First, Fast Healthcare Interoperability Resources (FHIR) provides a modular and extensible standard for organizing and querying clinical data. It supports the structuring of observations, imaging metadata, and laboratory results in a machine-readable format, enabling multimodal temporal correlation across systems. Second, blockchain-based digital wallets introduce decentralized identity and access control mechanisms that return data ownership to the patient while ensuring GDPR-compliant data traceability. These BioWallets allow individuals to store and control access to their longitudinal medical histories, including imaging records, annotations, and clinical summaries. Consent is managed through verifiable credentials and smart contracts, which are both transparent and auditable.

In this paper, we propose a novel framework that integrates these two technologies into a reusable, modular architecture for secure and patient-centric management of medical imaging data. The system is structured into four interoperable layers: (1) a FHIR-compliant data integration layer for ingesting and structuring imaging data and associated annotations; (2) a blockchain-based consent layer using Ethereum smart contracts; (3) a patient-facing BioWallet interface to manage access; and (4) an off-chain encrypted storage layer for image files and sensitive metadata.

To validate the framework, we implemented a proof-of-concept prototype using a curated dataset of 110 ARDS-COVID19 ICU patients. The dataset includes 220 anonymized chest radiographs and associated clinical variables such as ventilatory parameters and laboratory values. While the imaging files were available in PNG format, with cases available in DICOM format, allowing us to emulate DICOM-FHIR conversion and annotation strategies. Given the lack of structured reports, annotations were manually encoded into FHIR resources, enabling standardization for future reuse in AI pipelines. This step addresses one of the most critical bottlenecks in medical imaging: the absence of structured, reusable labels and metadata.

**Fig 1:**
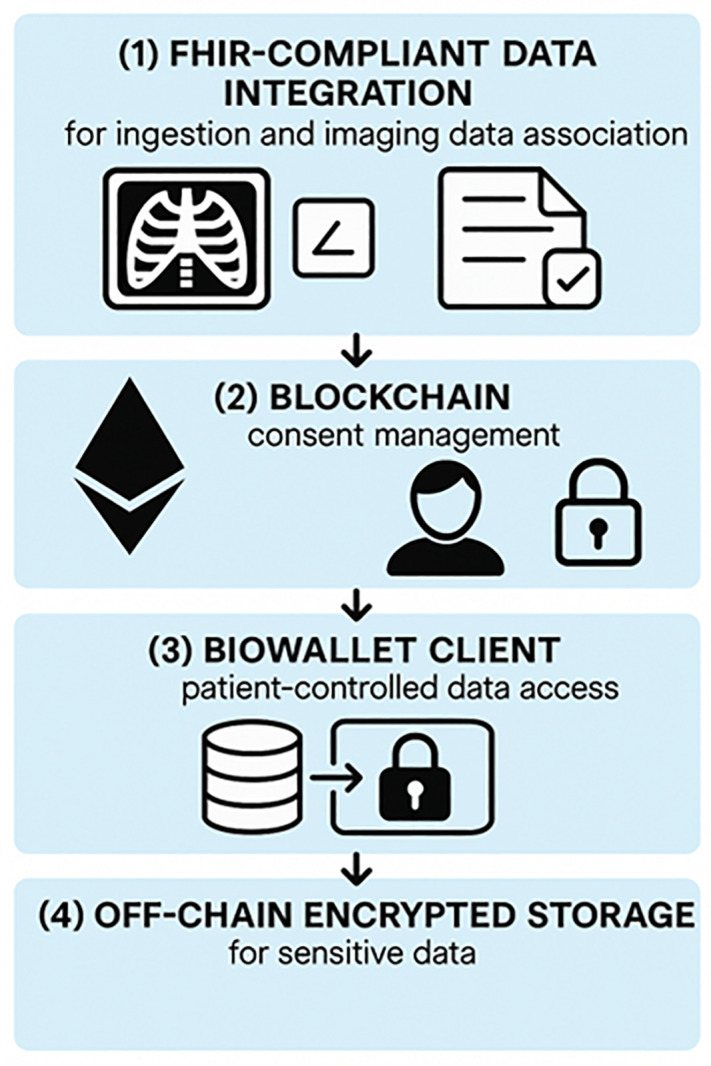
Patient-Centric Health Data Management Architecture. The architecture is composed of four functional components: (1) FHIR-compliant data integration for ingesting imaging data and annotations into structured formats; (2) blockchain-based consent management using smart contracts and decentralized identifiers (DIDs); (3) the BioWallet client for patient-controlled access and authorization; and (4) off-chain encrypted storage for safeguarding sensitive data. This pipeline supports secure, standards-based exchange of medical images and metadata.

Fig 1 illustrates the conceptual architecture of the proposed system, highlighting the four core layers that enable secure, interoperable, and patient-controlled management of clinical and imaging data. The model emphasizes the modular integration of blockchain technology, FHIR-based data standardization, smart contract–driven access control, and user-centric interfaces for consent and interaction through the BioWallet (Fig 1).

This leads us to our central research question:How can a blockchain-enabled, FHIR-compliant architecture integrating patient-controlled digital wallets facilitate secure, GDPR-aligned, and clinically useful access to medical imaging and clinical data for ICU patients with ARDS-COVID19?

The key contributions of this work are:

1. A reusable, patient-centric framework that combines FHIR and blockchain technologies to enable secure and interoperable sharing of medical images and annotations.
2. Implementation of decentralized consent management using verifiable credentials and smart contracts to ensure GDPR compliance and transparency.
3. Demonstration of how FHIR-based annotation structures can replace traditional free-text reports, facilitating AI-ready data labeling in critical care contexts.
4. Integration of multimodal, longitudinal ICU datasets for clinical monitoring and research purposes.
5. Validation through a proof-of-concept prototype simulating real-world ICU scenarios, including imaging ingestion, FHIR conversion, and decentralized access management.

While previous blockchain–FHIR frameworks such as FHIRChain [7], HealthChain [8], and TrialChain [9,10] demonstrated the feasibility of linking structured clinical data with blockchain-based consent or audit mechanisms, they typically treat imaging as a secondary data type and rely on institution-mediated authorization. BioWallet advances this line of work by placing imaging workflows at the center of the architecture and embedding patient-held decentralized identifiers and verifiable credentials as the primary mechanism for consent verification. Unlike prior models that focused mainly on provenance or cross-institution auditing, BioWallet operationalizes rapid, patient-mediated image retrieval suitable for ICU scenarios and integrates FHIR-native annotations to enable AI-ready reuse. Table 1 summarizes these distinctions, highlighting how the proposed framework extends earlier blockchain–FHIR integrations toward a fully implemented, imaging-first system validated under simulated critical-care conditions.

**Table 1:** Comparison of our work with existing literature.

Paradigm	Identity and consent model	Imaging support	Emergency access	Audit and provenance	Performance considerations	Reuse for AI (annotations/labels)	Typical deployment scope	Evidence reported
SMART on FHIR (OAuth2, app-to-EHR) [11]	Institution-mediated OAuth2 scopes; patient control indirect via portal/app	FHIR links to PACS via ImagingStudy/DiagnosticReport; DICOMweb integration varies by vendor	Usually ad hoc via institutional policy; not encoded in portable credentials	EHR-side logs; no immutable, cross-site audit	Good for metadata; raw DICOM retrieval depends on PACS/EHR connectors	Limited; annotations often remain unstandardized unless sites enforce structured reports	Single institution or tightly coupled networks	Large ecosystem adoption; limited imaging-centric evaluations
FHIRChain-style blockchain [7]	Blockchain-mediated consent; identity typically mediated via participating institutions	FHIR resources anchored on-chain; images off-chain; imaging treated as part of broader EHR exchange	Not typically modeled as pre-authorized, portable patient credentials	On-chain audit of access/consent events; tamper-evident	Latency acceptable for metadata; depends on ledger design and off-chain stores	Variable; imaging annotations not the primary focus	Multi-institution research data exchange	Conceptual and pilot studies; oncology decision-making trials cited
HealthChain and related frameworks [8,12]	Smart contracts enforce patient-granted access; identity varies by implementation	Structured clinical data; imaging findings/annotations supported conceptually	May be supported via contract logic; portability depends on identity layer	Immutable contract logs; provenance via hashes	Requires permissioned ledger and off-chain storage for scale	Possible via structured resources; not imaging-first	Research networks; pilots	Prototype-level reports; limited latency/usability metrics
TrialChain-style auditing [8,9]	Study- or site-mediated access; patient role less central	Focus on audit of imaging workflows; images off-chain; hashes on-chain	Not the primary design objective	Strong provenance of study artifacts and image events	Audit overhead low; does not optimize retrieval latency	Facilitates verifiable datasets; labels provenance-focused	Clinical trials, multicenter studies	Case studies of audited workflows
This work (BioWallet-imaging)	Patient-held DIDs/VCs in a digital wallet; smart contracts verify time-bound, scoped consent [7,13]	Imaging-first; DICOM→FHIR pipeline; images encrypted off-chain with FHIR pointers	Pre-authorized emergency access via wallet-issued credentials with explicit scope/expiry	Immutable, cross-site audit of access/consent; verified identifiers	Designed for ICU-like retrieval: permissioned ledger + off-chain storage for latency	Standardized annotations via FHIR resources to enable AI reuse	Cross-institution scenarios with patient-mediated exchange	Empirical latency reduction, consent enforcement success, and clinician usability collected in simulated ICU settings

The remainder of this paper is organized as follows: Section 2 presents related work on integrating blockchain and interoperability frameworks in healthcare, with a focus on data management, identity, and consent models. Section 3 describes the proposed methodology for integrating medical imaging datasets with blockchain-based digital health wallets, combining FHIR standards and decentralized identity mechanisms. Section 4 details the implementation process and presents the validation results using a real-world dataset of ARDS-COVID19 ICU patients. Section 5 discusses the security, privacy, and compliance aspects of the proposed framework. Finally, Section 6 concludes the paper and outlines directions for future work.

## 2. Literature review

Acute Respiratory Distress Syndrome (ARDS) is a severe, life-threatening lung condition that often arises in critically ill patients and was particularly prevalent during the COVID-19 pandemic. Chest imaging, especially radiography, is fundamental in diagnosing ARDS, assessing disease progression, and informing ventilatory strategies in Intensive Care Units (ICUs). In COVID-19-induced ARDS, frequent imaging is used to monitor pulmonary infiltrates, evaluate ventilator-associated complications, and guide therapeutic decisions. However, despite its centrality in clinical workflows, imaging data remains one of the least integrated and most fragmented data types in hospital information systems.

Timely access to imaging is often obstructed by the lack of interoperability between Picture Archiving and Communication Systems (PACS) and Electronic Health Records (EHRs). Even within the same hospital, radiographs may not be linked to structured clinical observations, and access is often mediated by institutional permissions or delayed by administrative bottlenecks. This fragmentation is particularly problematic in high-acuity settings such as ICUs and emergency departments, where clinical decisions are time-sensitive and depend on the immediate availability of imaging alongside laboratory and vital sign data.

Moreover, medical imaging datasets are typically stored in unannotated or inconsistently annotated formats, limiting their reuse in AI-based decision support systems or retrospective cohort analyses. Without structured annotation linked to clinical outcomes, the potential of imaging to support predictive modeling remains untapped. In the context of ARDS, the lack of semantically enriched, longitudinal imaging datasets poses a major barrier to understanding disease trajectories, optimizing treatment, and supporting ethically grounded research.

### 2.1. Data fragmentation and interoperability challenges in healthcare

Medical imaging is among the most siloed forms of data in healthcare. While picture archiving and communication systems (PACS) are designed for high-resolution storage and retrieval, they are not optimized for integration with clinical decision-making systems [14]. Imaging metadata are typically embedded in DICOM headers, which are not easily extracted or harmonized with structured clinical concepts such as diagnoses, treatments, or laboratory findings. This fragmentation hampers the construction of comprehensive, patient-centric views that are essential for both acute care and retrospective research [3,15].

Healthcare systems continue to face broader challenges related to data heterogeneity and the reliance on fragmented documentation practices. In high-acuity environments such as intensive care units (ICUs) and emergency departments, clinicians require timely access to longitudinal data, including imaging studies contextualized with vital signs, ventilation parameters, and laboratory results [16]. The lack of interoperability between EHRs, PACS, and laboratory systems often results in delays and incomplete decision-making support during critical episodes of care.

Interoperability standards such as Fast Healthcare Interoperability Resources (FHIR) have been developed to mitigate these issues by supporting the standardized exchange of structured health data, including imaging studies, through resources like ImagingStudy, DiagnosticReport, and Observation [17]. By converting radiological metadata into FHIR resources and linking them with clinical events, healthcare systems can achieve system-agnostic, near-real-time access to imaging data [14]. This is particularly relevant in conditions like COVID-19–induced ARDS, where radiographic imaging must be interpreted alongside contextual variables such as oxygenation indices, ventilator settings, and biomarkers like C-reactive protein (CRP) or D-dimer [16].

Even with DICOMweb (QIDO-RS/WADO-RS) and IHE profiles (e.g., XDS-I.b, MHD), harmonizing image references with FHIR resources and identity/authorization layers (e.g., SMART on FHIR/OAuth2) remains non-trivial across vendors and sites [11,17,18]. Technical barriers include the complexity of mapping heterogeneous DICOM metadata to FHIR structures, consistent terminology binding (SNOMED CT, RadLex, and LOINC for laboratories), and the absence of standardized workflows for annotating imaging studies for downstream reuse [16,17]. Standardization alone is insufficient: governance of consent, provenance, and cross-site authorization remains the critical bottleneck [19].

### 2.2. Blockchain applications in healthcare data management

Blockchain offers a decentralized, tamper-evident substrate for managing health data, addressing integrity, access control, and trust. Applied to medical imaging, blockchain can enable verifiable, patient-mediated access, transparent secondary use, and auditability across sites—capabilities that are particularly valuable in acute scenarios such as ARDS. Cryptographic hashes and immutable ledgers secure transaction logs, while images remain off-chain; this preserves privacy yet supports provenance and reproducibility. For example, architectures proposed during and after COVID-19 encrypted multimodal signals (including imaging) and logged access to clinical systems, reporting reductions in unauthorized access and improved auditability [20–24].

Traditional consent processes for image sharing are static and institution-bound. Smart contracts allow patients to define who can view or use their images, for how long, and under what conditions. In ARDS cases requiring second opinions or teleconsultations, smart contracts can automate access to prior imaging upon patient authorization, reducing delays while maintaining compliance (e.g., consent-workflow latency reductions have been reported) [22,23].

From a systems perspective, permissioned ledgers with off-chain storage are typically required to meet clinical performance targets for DICOM retrieval; public-chain designs without indirection are impractical for image payloads under ICU time constraints [24–26]. Most healthcare blockchain pilots centralize authorization via institutional gateways; few place patient-held credentials at the core of imaging access in acute care, which is the focus of our work. Blockchain traceability has also been explored for imaging device supply chains during crises; while orthogonal to data governance, it complements a provenance-aware ecosystem and is discussed briefly in [26,27].

Beyond healthcare-specific work, our design is also informed by the broader information-systems literature on blockchain implementation. Lu’s comprehensive review synthesizes operating mechanisms, core technologies, governance models, and common pitfalls in blockchain-based information systems, emphasizing the importance of permissioned deployments, off-chain storage, and organizational readiness for successful adoption [28]. Our architecture aligns with several of these recommendations: it adopts a permissioned ledger, minimizes on-chain payloads, separates audit from clinical content, and delegates node operation to institutional actors who already manage critical health information systems. At the same time, by centering patient-held decentralized identifiers and verifiable credentials in an imaging-first workflow, this work extends these general design principles into a high-acuity clinical domain that has been underrepresented in prior information-systems evaluations.

### 2.3. Decentralized identity, digital wallets, and consent management

Managing identity and consent for medical imaging is particularly challenging in ICUs, where decisions are time-sensitive, privacy risks are high, and GDPR compliance is imperative [3,29]. Decentralized identifiers (DIDs) and verifiable credentials (VCs) (W3C) enable patient-held, cryptographically verifiable identities and consent artifacts that are portable across institutions [30–33]. In our model, the BioWallet stores VCs for patients and authorized clinicians; the wallet presents VCs, smart contracts verify scope (for example, modality, study date, or purpose), and DICOM objects remain encrypted off-chain with FHIR pointers. Emergency access can be modeled via pre-issued VCs or policy-bound contracts with explicit expiry and scope, enabling immediate retrieval even when patients are incapacitated, while preserving accountability [33,34].

Prior work established feasibility for DID/VC-based consent and cross-institution sharing of imaging/EHR data [10,15,35]. These systems demonstrate the building blocks; our contribution operationalizes DID/VC-mediated consent specifically for imaging under ICU-like time constraints and reports empirical usability and latency outcomes.

### 2.4. Integrating blockchain and FHIR for clinical research enablement

FHIR provides ImagingStudy, DiagnosticReport, and Media to represent radiological metadata and link images with observations, procedures, and outcomes. The remaining challenge in multi-stakeholder research settings is secure, auditable access across sites. Early blueprints such as the ONC Blockchain Challenge (MIT) whitepaper articulated decentralized consent and provenance for health data and prefigured patient-mediated exchange [36]. Reviews of blockchain in medical imaging synthesize open issues around consent, identity, performance, and reproducibility [37], while broader analyses of blockchain for health data exchange underscore governance and auditability gaps [7].

FHIRChain-style models integrated HL7 FHIR with blockchain to support diagnostic data exchange across institutions; in oncology decision-making trials, imaging metadata were linked to patient identities, enabling transparent consent tracking and secure access [7]. HealthChain further explored smart-contract enforcement of patient-granted access to structured data, including radiographic findings and annotations [8]. Compared with SMART on FHIR (OAuth2 scopes mediated by institutional EHR apps), blockchain-anchored DIDs/VCs support patient-held credentials with immutable audit trails and cross-site portability [11,32].

For large-scale imaging studies, permissioned platforms map imaging and trial metadata to FHIR resources and use smart contracts to enforce protocol compliance and track access queries across research sites [13]. TrialChain illustrates blockchain-based auditing for imaging workflows, ensuring verifiable provenance for every accessed or annotated diagnostic image [9].

Recent studies have expanded these paradigms by coupling blockchain infrastructures with artificial intelligence and large language models (LLMs) to support privacy-preserving clinical decision systems. For instance, LLM-assisted blockchain frameworks have been explored for secure federated analytics and explainable diagnostics in multi-hospital environments [38,39]. Similarly, hybrid architectures combining decentralized identity with edge-based AI inference demonstrate the growing convergence between trustworthy data exchange and adaptive intelligence in healthcare [40,41]. These developments reinforce the importance of integrating standardized interoperability (FHIR) with blockchain-based governance as foundational enablers of ethical, AI-ready data ecosystems.

Therefore, the literature supports a convergence of FHIR and blockchain as a scalable, ethically aligned path for imaging governance in high-acuity and research-intensive settings. Distinct from prior work, our approach is imaging-first, centers patient digital wallets (DID/VC) for pre-authorized emergency access, standardizes DICOM→FHIR annotations for AI reuse, and reports empirical latency and consent-enforcement under ICU-like conditions. In summary, the literature converges on the need to couple interoperable representations of imaging data with trustworthy consent and audit mechanisms, yet existing approaches tend either to prioritize general EHR exchange over imaging workflows or to emphasize auditing without optimizing retrieval performance. The proposed framework positions itself as imaging-first by standardizing DICOM-to-FHIR mappings for radiographs, placing patient-held DIDs and verifiable credentials at the center of access control, and operationalizing pre-authorized emergency access in a manner compatible with ICU time constraints. By separating on-chain consent verification and audit from encrypted off-chain storage, it achieves practical latency while preserving cross-site traceability. Unlike prior paradigms that rely on institution-mediated authorization or treat imaging as a secondary data type, this work demonstrates a wallet-centered model that supports AI-ready annotations and reports empirical evidence on latency, consent enforcement, and clinician usability.

## 3. Methodology

This study applies the Design Science Research Methodology (DSRM) to design, implement, and evaluate a secure and interoperable framework for patient-mediated access to imaging and clinical data. The six DSRM stages—problem identification, objectives, design and development, demonstration, evaluation, and communication—structure the work, with emphasis on the artifact’s operational details and its alignment with clinical constraints.

### 3.1. Problem and objectives

Intensive care settings require timely, trustworthy access to longitudinal data across heterogeneous systems. In practice, hospitals continue to face four persistent limitations: fragmentation across PACS, EHR, and laboratory information systems; consent processes that are manual and institution-bound; limited mechanisms for patient control over data use; and a lack of standardized annotations that would enable secondary uses such as AI development. The objective of this work is to produce an architecture that exposes imaging and key clinical variables through FHIR resources, enforces patient-mediated and revocable consent using decentralized identifiers (DIDs) and verifiable credentials (VCs) verified by smart contracts, and maintains provenance and latency characteristics appropriate for time-critical care.

### 3.2. Artifact overview

**Fig 2:**
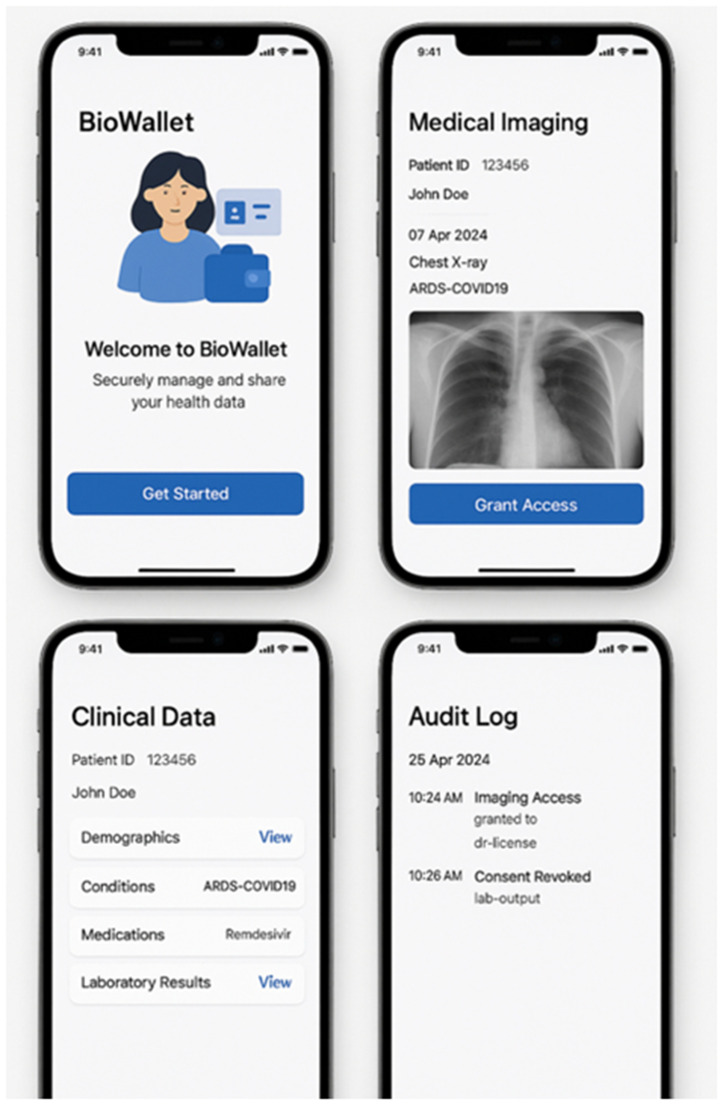
Workflow for transforming medical imaging files into standardized FHIR resources for AI applications. Imaging files in DICOM or PNG format are converted through a DICOM-to-FHIR process, followed by manual annotation. The resulting FHIR resources enable standardized data representation for future reuse in AI pipelines. This framework was tested in a simulated ICU setting where clinicians and researchers queried longitudinal datasets based on temporal alignment between imaging, ventilation, and laboratory results. Access control was governed through the BioWallet, allowing simulated patients to grant or revoke access to subsets of their data. These validation activities highlight the feasibility of integrating such a system into clinical research workflows without compromising data sovereignty or privacy.

The artifact is organized into four interacting layers connected by standard APIs. The data layer curates and anonymizes an ICU cohort. A FHIR integration layer publishes the structured representation of imaging and clinical variables. A consent and access layer runs on a permissioned Ethereum network, where smart contracts verify policy compliance based on DIDs and VCs. Finally, a storage and client layer keeps DICOM objects in encrypted off-chain storage and exposes a patient-facing BioWallet that mediates consent and access. Fig 2 depicts the layers and their interfaces.

To facilitate standardized processing, PNG files were programmatically converted into DICOM format using metadata templates extracted from the available native DICOM cases. This conversion enabled uniform handling across the imaging dataset and ensured compatibility with downstream DICOM-to-FHIR transformation tools. The complete workflow—from PNG conversion to DICOM, FHIR resource generation, and manual annotation—is illustrated in Fig 2, which summarizes the steps required to standardize imaging data for AI reuse, while maintaining alignment with FHIR-based interoperability and patient consent governance mechanisms.

### 3.3. FHIR data integration pipeline

The dataset comprises 110 patients admitted to the ICU with ARDS-COVID19, each with two chest radiographs. When native DICOM was unavailable, PNG images were converted to DICOM using template metadata derived from native studies to ensure uniform handling. All images were anonymized by removing direct identifiers in DICOM headers. Pseudonymous patient identifiers were generated and applied consistently to imaging and clinical tables to preserve longitudinal linkage while protecting privacy.

Clinical and imaging information was then mapped to FHIR resources hosted on a HAPI-FHIR server. ImagingStudy resources were derived from DICOM header fields. StudyInstanceUID, SeriesInstanceUID, and SOPInstanceUID were mapped to the appropriate ImagingStudy identifiers and nested series and instance elements; modality and acquisition timestamps were recorded to support temporal queries; and the study start was recorded in the ImagingStudy.started element. Each imaging event was represented by a DiagnosticReport that referenced the corresponding ImagingStudy and, where appropriate, encoded structured conclusions using RadLex or SNOMED CT. Observations captured ventilatory parameters such as PEEP and FiO2, oxygenation indices including the PaO2/FiO2 ratio, and laboratory biomarkers such as CRP and D-dimer using LOINC and SNOMED CT codes. Provenance resources tracked transformations such as PNG-to-DICOM conversion, identifying the agents and timestamps involved. Patients and clinicians were represented by Patient and Practitioner resources, and references were established so that DiagnosticReport entries pointed to the relevant Observations and ImagingStudy for the same encounter and time window. Access to structured data used standard FHIR REST search parameters, including chains by patient and date. Pixel data were not stored in FHIR; instead, ImagingStudy contained resolvable pointers to off-chain objects, which were retrieved through signed URLs or WADO-RS endpoints only after consent verification, as described in Section 3.4.

To contextualize the cohort, we report patient-level demographics and key clinical characteristics in Table 2. Among 110 ICU patients with ARDS-COVID19 treated in Portugal, the median age was 62 years (IQR 54–71), and 38% were female and 62% male. The most frequent comorbidities were hypertension (56%) and diabetes (28%). The median APACHE II score at ICU admission was 17 (IQR 13–22). Based on Berlin ARDS criteria, 22% of patients had mild, 48% moderate, and 30% severe ARDS. The median PaO₂/FiO₂ ratio at the time of the first radiograph was 132 (IQR 95–176). Eighty-two percent of patients were invasively ventilated, and 18% received noninvasive ventilation. The median ICU length of stay was 14 days (IQR 9–22), with an ICU mortality rate of 32%. Two radiographs per patient were included (total 220); the first was acquired a median of 8 hours after ICU admission and the second after 72 hours. Of the 220 images, 65% originated as PNG and 35% as native DICOM prior to standardization.

**Table 2:** Cohort overview (ICU ARDS-COVID19).

Characteristic	Value
N (ICU ARDS-COVID19 patients)	110
Age, years — median (IQR)	62 (54–71)
Sex — female/male	38% / 62%
APACHE II at admission — median (IQR)	17 (13–22)
Comorbidities — hypertension	56%
Comorbidities — diabetes	28%
ARDS severity (Berlin) — mild/moderate/severe	22% / 48% / 30%
Ventilation mode at first film — invasive/noninvasive	82% / 18%
PaO₂/FiO₂ at first film — median (IQR)	132 (95–176)
ICU length of stay, days — median (IQR)	14 (9–22)
ICU mortality	32%
Images per patient	2 (total 220 radiographs)
Timing of films vs ICU admission — median hours (film 1 / film 2)	8 / 72
Image origin — PNG / DICOM	65% / 35%

For the validation experiments, we did not expose the original cohort in full fidelity. Instead, we generated synthetic ICU cases derived from the curated ARDS-COVID19 dataset. Pixel data consisted of the anonymized radiographs described above, while clinical context variables (e.g., ventilatory parameters, laboratory values, and timestamps) were transformed to reduce re-identification risk. Specifically, we (i) replaced all direct identifiers with pseudonyms, (ii) jittered time stamps within clinically plausible windows (±2–4 hours for imaging events, ± 12–24 hours for laboratory measurements) while preserving intra-patient ordering, (iii) added small, bounded noise to continuous variables (for example, ± 5–10% around the original value for PaO₂/FiO₂ or CRP) and then re-clipped to physiological ranges, and (iv) stratified sampling by ARDS severity so that the synthetic cases preserved the original distribution of mild, moderate, and severe ARDS. These transformations maintained realistic joint distributions for evaluation while preventing reconstruction of individual trajectories.

### 3.4. Consent and access control with Ethereum smart contracts and DIDs/VCs

Identity and authorization were modeled using DIDs for patients and clinicians and VCs to encode roles and permissions. The BioWallet stored the subject’s cryptographic keys, DIDs, and VCs and never exported private keys outside the device. The blockchain stored only hashes and minimal metadata necessary for audit and policy verification, such as the hash of a consent record, the declared scope, and timestamps. Clinical content remained off-chain in encrypted object storage. Access URLs were short-lived and generated only after successful policy checks.

In our architecture, the blockchain records only tamper-evident audit and consent artifacts (hash of consent, scope, timestamps). No clinical content is stored on-chain. Imaging pixels remain in encrypted off-chain storage, and clinical context is exposed via FHIR resources. As a result, clinical corrections (for example, fixing an erroneous observation or report) are made in the FHIR/EHR layer through standard versioning and Provenance, while the chain retains an immutable trail of access and consent decisions. This preserves auditability without impeding legitimate rectification of patient records.

Consent was represented as a structured object that specified the patient’s DID, the requester’s DID, the scope of access in terms of resource types and constraints such as modality or date range, the declared purpose such as treatment or research, and the validity interval including not-before and expiry times. The patient created or approved consent in the wallet, which posted a signed descriptor to off-chain storage and wrote its hash on-chain through a contract transaction.

Read access proceeded through a deterministic flow. A clinician application initiated a request for a specific patient, date range, and study type. The application presented its VCs together with the requested scope to a policy gateway. The gateway invoked the smart contract to verify the clinician’s identity and role, the existence of a matching and non-revoked patient consent, the requested scope and purpose, and the validity interval. If the check succeeded, the contract emitted an audit event with hashed identifiers and the verified scope, and the gateway minted a short-lived access token and generated signed URLs to the required off-chain objects. The client then retrieved pixel data via WADO-RS or direct HTTP using those URLs. If any check failed, the contract wrote a denial event to the ledger and no token was issued. Revocation was immediate and patient-driven: submitting a revocation transaction caused subsequent verification attempts to fail until a new consent was established. Emergency access was modeled as a pre-authorized credential with explicit scope and short validity, enabling immediate access when patients were incapacitated while preserving an auditable trail.

To ensure fine-grained and auditable enforcement, the smart contract encodes access rules as on-chain functions that validate decentralized identifiers, verifiable credentials, and consent descriptors. Each consent record contains: (1) the patient’s DID, (2) the requester’s DID and role, (3) the allowed resource scope (for example, ImagingStudy or DiagnosticReport), (4) a purpose tag (treatment, research, emergency), and (5) validity timestamps (notBefore, expiry). During access verification, the contract retrieves the requester’s VC hash, matches it against the consent scope and time window, and emits an event only if all checks pass. Revoked consents are marked inactive by setting their status flag to false, preventing further matches. The pseudocode below summarizes the core logic:

function verifyAccess(

bytes32 patientDID,

bytes32 requesterDID,

string memory resourceType,

string memory purpose,

uint256 currentTime

 ) public view returns (bool authorized) {

Consent memory c = consentRegistry[patientDID][requesterDID];

require(c.active, “Consent revoked or not found”);

require(currentTime >= c.notBefore && currentTime <= c.expiry, “Expired or not yet valid”);

require(compareStrings(resourceType, c.scope) && compareStrings(purpose, c.purpose), “Scope or purpose mismatch”);

require(matchVC(requesterDID, c.allowedRoles), “Role not authorized”);

return true; // Emit audit event off-chain

}

In the deployed contract, matchVC verifies that the requester’s DID corresponds to a valid verifiable credential issued by a trusted authority and that the declared role (e.g., radiologist, intensivist) is permitted within the consent scope. Successful verifications trigger an on-chain event (AccessGranted) containing hashed identifiers and a timestamp, while failed attempts generate AccessDenied logs for audit. This minimal logic ensures transparent, deterministic, and non-repudiable enforcement of consent policies.

Threats in this layer were handled through DID-based challenge–response and biometric unlocking in the wallet to mitigate spoofing, immutable contract events that linked to consent hashes to address tampering and repudiation, and a strict reference architecture in which only hashes and minimal descriptors appeared on-chain while encrypted payloads remained off-chain. Signed URLs expired shortly after issuance to reduce the exposure surface.

To preserve usability, gas costs and transaction frequency were explicitly constrained. The permissioned Ethereum network (Hyperledger Besu, IBFT) operated with a fixed low gas price that is fully absorbed by the operating institution; neither patients nor clinicians are exposed to gas fees or blockchain-specific concepts in the user interface. The smart contracts were designed so that only a minimal set of events—consent creation, revocation, and access audit logs—generate on-chain transactions. All clinical data access and FHIR queries occur off-chain once the contract has emitted an authorization event. This design bounds the number of transactions per user session while preserving a complete, tamper-evident audit trail.

### 3.5. Prototype implementation

The FHIR stack used HAPI-FHIR (R4) with terminology services for SNOMED CT and LOINC and with ImagingStudy, DiagnosticReport, and Observation search optimized for time-series queries. The ledger was a permissioned Ethereum network implemented with Hyperledger Besu and IBFT consensus. Smart contracts were written in Solidity with event topics indexed to facilitate audit retrieval. Storage consisted of an encrypted IPFS gateway backed by an S3-compatible object store; upon successful contract verification, a policy service generated time-limited, scope-bound URLs. The wallet application was developed in Flutter and implemented DID methods compatible with did:ethr and did:key, handled VCs according to the W3C data model, and stored keys in a secure enclave or keystore with biometric unlock. A gateway service mediated between FHIR search, smart contracts, and storage, exposing a single endpoint to clinical applications so that clients did not implement contract logic directly. To avoid excessive transaction overhead, the gateway aggregates multiple image accesses performed under the same verified consent into a single on-chain audit event per session, while keeping a more granular off-chain log for local governance; this form of transaction batching reduces blockchain interaction without weakening auditability.

### 3.6. Demonstration, evaluation, and communication

The demonstration reproduced ICU-like workflows in which external clinicians retrieved patient imaging and contextual observations after patient-granted consent. The end-to-end path exercised FHIR search, smart-contract verification, audit logging, signed-URL issuance, and WADO-RS retrieval. The evaluation protocol, detailed in Section 4, measured access latency from authentication to image availability, consent-verification time from contract call to response, success and denial rates, and audit completeness; it also assessed human-factors outcomes including task completion, usability, and perceived security. Security analysis followed STRIDE across wallet, gateway, contracts, FHIR services, and storage components and included negative tests with rate-limiting and envelope encryption. The system was deployed in a cloud sandbox configured to emulate hospital network boundaries. Resource definitions and dataset organization adhered to FAIR principles. The communication phase includes publication of the framework and validation results and preparatory work for pilots integrating national electronic identity and wallet services to support cross-institution exchange.

## 4. Implementation and results

### 4.1. Scenario overview

To assess applicability and performance in critical care, we implemented a validation scenario that reproduces key workflows from an intensive care unit during the COVID-19 surge. The goal was to determine whether the system could provide timely, secure, and consent-driven access to radiological images together with clinically relevant annotations. We focused on end-to-end retrieval latency, correctness and speed of consent enforcement through the BioWallet, and the user experience of healthcare professionals operating under time pressure. The core task required a clinician without institutional PACS credentials to retrieve a patient’s chest radiographs and the temporally aligned clinical context needed for an urgent decision. All data used during the study were anonymized prior to ingestion. Radiographs were available as DICOM objects referenced by FHIR ImagingStudy and linked to DiagnosticReport and Observation resources. Access was governed by smart contracts and activated by verifiable credentials stored in the patient’s BioWallet.

### 4.2. Setup

Experiments were conducted in a sandboxed testbed that mirrored a hospital network perimeter. The stack comprised a HAPI-FHIR server for structured metadata, a permissioned Ethereum network (Hyperledger Besu) for consent verification and audit logging, and an encrypted IPFS-backed object store for DICOM files. Participants interacted using a mobile BioWallet application implemented in Flutter with biometric unlock and DID-based identity.

Three practicing clinicians were recruited to perform retrieval tasks on synthetic cases drawn from the curated ARDS-COVID19 cohort. Each case included two chest radiographs and associated ICU variables, and each participant completed the same sequence of tasks: authenticate, request patient-mediated consent, retrieve the most recent imaging study together with the temporally corresponding observations, and verify the linkage between the DiagnosticReport and its ImagingStudy. To exercise the denial path and the audit trail, we injected controlled unauthorized requests in two forms: role-mismatched access from a valid identity and access using an expired or revoked consent token. A separate scalability check used five concurrent client sessions to probe the effect of simultaneous requests on latency; these concurrent sessions did not change the number of clinician participants.

**Fig 3:**
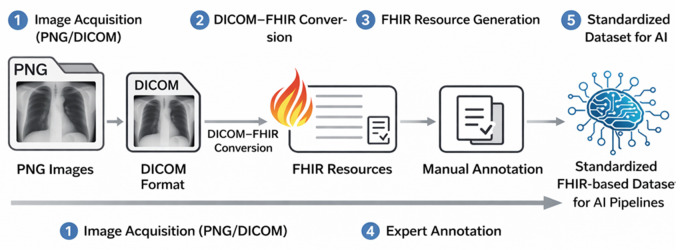
Mockups for the application.

To test the system's resilience and access control rigor, predefined unauthorized access attempts were introduced. These simulated both insider misuse and external threats, allowing for an assessment of the smart contract layer's responsiveness and the audit logging system’s fidelity. The Fig 3 presents the mockups of the application:

### 4.3. Metrics and data collection

System performance and user experience were measured in a manner intended to be reproducible. End-to-end access latency was defined as the interval between successful user authentication and the moment when both the radiographic pixel data and the time-aligned observations were available to the client. This measurement included FHIR query time, on-chain consent verification, audit logging, signed URL generation, and image retrieval via WADO-RS or HTTPS. Timestamps were recorded at the gateway and at the client with synchronized clocks and stored in an append-only log.

Consent-verification time was defined as the interval between the contract call invoked by the policy gateway and the reception of the on-chain receipt emitted by the contract, and thus excluded network transfer of pixel data. Retrieval success and denial events were counted at the gateway and confirmed against the contract event stream. Audit completeness was assessed by reconciling gateway logs with emitted contract events across all authorized and denied requests.

Clinician-reported usability and perceived security were collected immediately after each task run using a brief, self-administered questionnaire on the study device. The instrument comprised two five-point Likert items (1 = strongly disagree, 5 = strongly agree): ease of use (“I was able to complete the imaging retrieval tasks without unnecessary effort”) and perceived data security (“I am confident that the system enforces patient consent and protects sensitive data”). Each clinician completed two runs; for analysis, item scores were first averaged within participant across runs, then aggregated across the three clinicians to yield the group mean. Likert responses were treated as approximately interval-scaled, and dispersion was summarized by the standard deviation across participants. Open-ended prompts captured qualitative comments on provenance displays, consent workflow clarity, and any points of friction. All responses were complete and non-identifying; no imputation was required.

### 4.4. Results

End-to-end access latency was measured from the moment the clinician completed authentication to the moment the radiographic pixel data and time-aligned observations were fully available in the client viewer. For the baseline, we replicated the conventional EHR+PACS workflow used in the host institution: (1) clinician authentication via institutional single sign-on, (2) manual navigation to the patient record, (3) consent confirmation following local procedures, and (4) manual retrieval of the most recent chest radiograph via the PACS viewer. Under this baseline workflow, average end-to-end access time over 30 repeated trials (3 clinicians × 10 cases) was 12.8 ± 2.1 seconds.

Using the BioWallet architecture under the same network conditions and for the same cases, mean access time was 4.2 ± 0.7 seconds, corresponding to a 67% reduction in latency. In this setting, the pipeline consolidates consent verification, FHIR search, and DICOM retrieval into a single gateway-mediated request, eliminating manual cross-system navigation. Unauthorized access attempts were blocked in all injected test cases (100% denial success), and all authorized or denied requests were visible in the audit stream.

Consent verification by the smart-contract engine completed in a mean of 1.73 seconds across all authorized requests. No contract-level failures were observed. In the five simulated unauthorized attempts, comprising three insider-style role mismatches and two cases of expired or revoked consent, access was denied and each event was recorded on-chain with hashed identifiers, timestamp, device signature, and requested scope. An audit of 25 authorized transactions showed a one-to-one correspondence between gateway logs and contract events, with no missing entries.

The concurrency probe using five simultaneous client sessions produced an 11.5% increase in average latency relative to single-session trials. This change was consistent with expectations for the architecture, which separates concerns between the FHIR server, the on-chain verification step, and off-chain object retrieval.

Clinician-reported usability and perceived security were favorable. The mean score for ease of use was 4.6 (SD 0.58) and for perceived data security was 4.8 (SD 0.41). These values were computed by averaging, for each clinician, the two post-run ratings for each item and then averaging those per-participant means across the three clinicians. Free-text comments highlighted explicit provenance links between DiagnosticReport and ImagingStudy, transparency of the consent interface, and clear denial feedback for out-of-scope requests.

To contextualize performance, BioWallet’s latency and access-control results were benchmarked against a conventional hospital PACS workflow used as the baseline. The baseline represented the standard process of clinician authentication via institutional single sign-on, followed by manual consent verification and DICOM retrieval through the PACS viewer. Average end-to-end image-access time in this setup was 12.8 ± 2.1 seconds. Under identical network conditions, the BioWallet prototype achieved a mean retrieval time of 4.2 ± 0.7 seconds, corresponding to a 67 percent reduction in latency. Unauthorized-access attempts were blocked in all test cases (100 percent denial success), whereas audit visibility—defined as the proportion of access events automatically recorded and retrievable from logs—rose from 42 percent in the baseline to 100 percent with BioWallet. These results align with and quantitatively extend earlier blockchain-FHIR prototypes such as FHIRChain [7] and HealthChain [8], which primarily reported feasibility without detailed latency measurements. The observed performance advantage therefore derives from pre-verified credentials, off-chain encrypted storage, and the deterministic smart-contract validation pathway that replaces multi-step manual authorization.

### 4.5. Key observations and implications

The study indicates that combining a FHIR-centric representation of imaging metadata with wallet-mediated consent and smart-contract enforcement is feasible and advantageous in time-sensitive settings. Standardized resources and terminology services supported consistent linking between radiographs and contemporaneous observations, while the BioWallet allowed patient-centric, revocable permissions to govern cross-institution access. The separation of functions—encrypted off-chain storage for pixel data, FHIR for indexing and discovery, and a permissioned ledger for policy checks and audit—reduced latency without compromising auditability. An important clinical insight is that pre-authorized emergency credentials enable immediate access when patients are incapacitated, with a verifiable and immutable trail of who accessed what and when. This property directly addresses long-standing gaps in PACS-centric workflows, where consent traceability and cross-site audit are limited. Practically, this means immutability strengthens auditability (who, what, when) without constraining correction of clinical content, which continues to follow standard EHR/FHIR governance and versioning.

The study indicates that combining a FHIR-centric representation of imaging metadata with wallet-mediated consent and smart-contract enforcement is feasible and advantageous in time-sensitive settings. Standardized resources and terminology services supported consistent linking between radiographs and contemporaneous observations, while the BioWallet allowed patient-centric, revocable permissions to govern cross-institution access. The separation of functions—encrypted off-chain storage for pixel data, FHIR for indexing and discovery, and a permissioned ledger for policy checks and audit—reduced latency without compromising auditability. An important clinical insight is that pre-authorized emergency credentials enable immediate access when patients are incapacitated, with a verifiable and immutable trail of who accessed what and when. This property directly addresses long-standing gaps in PACS-centric workflows, where consent traceability and cross-site audit are limited. Practically, this means immutability strengthens auditability (who, what, when) without constraining correction of clinical content, which continues to follow standard EHR/FHIR governance and versioning. The quick access observed in our evaluation reflects the system’s ability to minimize verification and retrieval delays through its hybrid architecture and consent automation, allowing authorized clinicians to obtain imaging data efficiently while preserving full security and governance controls.

The performance gains, together with high perceived security, suggest that the architecture can shorten decision cycles for common ICU actions while improving accountability. Beyond direct care, the same pipeline provides a foundation for ethically aligned secondary uses, since annotated imaging can be made available for federated learning without exporting raw data outside institutional boundaries. The modular design, which already accommodates chest radiography, extends naturally to CT and MRI by preserving off-chain storage and reference-based retrieval, and by maintaining the same consent and audit semantics.

A key limitation of this evaluation is the small number of end users: only three practicing clinicians participated in the validation tasks, and all interactions occurred in a sandboxed environment rather than in a live ICU. As such, the reported latency, consent-enforcement success, and usability scores should be interpreted as feasibility indicators rather than statistically generalizable estimates. In line with the Design Science Research Methodology underpinning this work, our focus was on demonstrating the viability and clinical plausibility of the architecture. Future work will include multi-site deployments with larger and more diverse clinician cohorts, background traffic representative of production hospital networks, and standardized usability instruments to quantify adoption barriers and workflow impact.

## 5. Security analysis

Securing radiological data poses unique challenges in digital health infrastructures, as imaging files are often large, sensitive, and rich in identifiable features. The proposed architecture addresses these challenges through a multi-layered security framework specifically adapted to imaging workflows in intensive care environments. The architecture integrates off-chain encrypted image storage, decentralized identity (DID) mechanisms, and smart contract-governed access controls to ensure that imaging data remain both private and traceable throughout their lifecycle.

One of the core design principles was the decoupling of raw imaging data from on-chain transactions. DICOM files, containing not only pixel data but also embedded metadata in headers, are stored in a distributed encrypted repository, ensuring that sensitive content never traverses the blockchain. Instead, access is mediated via FHIR-compliant ImagingStudy and DiagnosticReport resources that point to secure storage locations. Smart contracts record and enforce access decisions, while all retrieval actions—whether successful or denied—are immutably logged.

We deliberately decouple audit from content. The ledger’s immutability provides non-repudiable evidence of consent verification and access events; it does not lock clinical values. Corrections to imaging reports or observations are issued as new resource versions in FHIR with Provenance links, while the blockchain continues to log who accessed which objects under what policy. This separation meets the need for traceability in adversarial or cross-site scenarios while remaining compatible with clinical error correction and GDPR rights such as rectification and revocation of consent, since only access events and consent hashes are immutable and payloads remain off-chain and replaceable.

Access control is implemented through verifiable credentials cryptographically tied to DIDs, issued to both patients and authorized healthcare professionals. These credentials must be presented through the BioWallet interface to initiate any retrieval operation. For imaging specifically, credentials include metadata-based constraints (e.g., modality type, study date) ensuring fine-grained access policies. This is particularly important for ICU use cases, where radiological assessments (e.g., chest X-rays) must be rapidly shared across departments without compromising patient consent or privacy.

To evaluate the architecture’s threat resilience, a formal STRIDE-based threat modeling analysis was conducted across components managing image metadata (FHIR server), storage (IPFS), access logic (smart contracts), and user interface (BioWallet). Spoofing risks in image access were mitigated using biometric authentication combined with DID resolution. Tampering was addressed by ensuring that any reference to image location or annotation history is cryptographically secured and auditable via blockchain logs. Repudiation concerns—especially in image annotation and access—were countered by ensuring every action is non-repudiable and linked to verified identifiers. Data leakage risks were minimized by never exposing DICOM content through APIs directly, and instead using signed requests that only resolve under consent-confirming conditions.

Synthetic attack scenarios focused on imaging data were integrated into the evaluation phase. These included attempts to access unauthorized imaging studies, modify annotation-linked metadata, and spoof user identities to bypass radiology-based access constraints. All such attempts were blocked at the contract or DID validation layer, and corresponding access requests were logged with cryptographic integrity for retrospective review. These results demonstrate the system’s capacity to maintain both image confidentiality and provenance tracking, which are essential not only for clinical trust but also for AI readiness in secondary data use.

Importantly, this framework anticipates regulatory and ethical obligations surrounding image reuse [12,42,43]. With the inclusion of dynamic consent mechanisms, patients can revoke access to previously shared imaging studies, ensuring that longitudinal control remains with the data subject—even in emergency contexts. This aligns with GDPR provisions for data subject rights, and with ethical best practices for image-based AI development.

While current simulations confirm the system’s robustness under test conditions, further security evaluations in live hospital settings are necessary [12,42,43]. Future developments should include penetration testing focused on the imaging pipeline, adversarial probing of access control boundaries, and implementation of privacy-preserving technologies such as zero-knowledge proofs to further limit unnecessary exposure of imaging identifiers or metadata. Still, the present analysis affirms that the architecture meets the stringent requirements for secure and auditable imaging access in critical care environments, where trust, speed, and privacy must coexist.

Although imaging data remain stored within institutional repositories, this hybrid approach is intentional and compliant with clinical and regulatory constraints. Decentralization in this framework refers primarily to the distribution of trust and consent control through verifiable credentials and smart contracts rather than to the physical dispersion of imaging files. Institutional gateways act as trusted nodes that mediate access between encrypted storage and authorized users, ensuring low latency and auditability while maintaining alignment with health data–protection requirements. This design choice balances the technical feasibility of blockchain with the operational realities of hospital infrastructure.

Furthermore, this hybrid design introduces explicit trust assumptions. While the ledger decentralizes consent verification and audit, institutional gateways remain trusted components for mediating access between the blockchain, FHIR server, and encrypted storage. A fully on-chain approach could in principle reduce reliance on gateways but is infeasible in our context because of DICOM file sizes, privacy constraints, and the need to support correction of clinical content. Instead, we accept a controlled degree of institutional trust in exchange for practical latency, compatibility with existing infrastructures, and the ability to deploy incrementally within hospital environments.

Under this model, several attack vectors must be considered. A compromised gateway could attempt to bypass contract checks or serve unauthorized images; we mitigate this risk by requiring that every successful access be corroborated by an on-chain AccessGranted event, by keeping detailed off-chain logs for cross-checking, and by isolating gateway services within the hospital perimeter with strict authentication and rate limiting. Collusion between institutional nodes could, in theory, weaken consensus; the permissioned network therefore assumes a limited, vetted set of operators and can be complemented by external audits of contract events. At the edge, theft of a patient’s or clinician’s wallet keys could enable fraudulent consent or access; here we rely on secure enclave/keystore storage, biometric unlocking, and the ability to revoke credentials and DIDs. Finally, metadata leakage remains a concern even when pixels are encrypted; we minimize exposed identifiers in URLs and plan to explore additional techniques such as short-lived opaque references and, in future work, zero-knowledge proofs to further constrain what must be revealed during policy enforcement.

Making these trade-offs explicit clarifies that decentralization in our framework primarily concerns consent and auditability, while storage and network operations remain anchored in clinical institutions. We argue that, given current technical and regulatory constraints, this balance offers a pragmatic path to improving trust and transparency in imaging data governance without overpromising full infrastructural decentralization.

## 6. Conclusion

The validation of this architecture underscores the transformative potential of patient-centered, blockchain-enabled health data systems, particularly for imaging data governance. The framework demonstrates that annotated radiological assets can be ingested, stored, accessed, and traced through a secure, standards-compliant pipeline, thereby laying the groundwork for both improved patient care and accelerated secondary uses such as AI-based diagnostics and retrospective clinical studies.

A central implication of this work is the redefinition of data ownership and access rights. By integrating verifiable credentials and decentralized identifiers, the architecture enables patients to control who can view their imaging data and under what conditions. This model proves especially valuable in intensive care settings where patients may be incapacitated and rapid clinical decisions are necessary. Pre-authorized consents stored in the BioWallet allow for cryptographic validation of access in real time, ensuring that clinicians can retrieve radiological images without procedural delays or compromising privacy. This governance model aligns with evolving legal frameworks such as the General Data Protection Regulation and anticipates interoperability with upcoming digital identity initiatives, including the European Digital Identity Wallet.

The clinical benefits of reduced latency are also clear. In scenarios where time-sensitive access to imaging data informs decisions on ventilation or escalation of care, the ability to cut retrieval time by over 60 percent represents a substantial gain in responsiveness. Beyond speed, the structured representation of radiological information using FHIR resources such as ImagingStudy and DiagnosticReport ensures that clinicians receive not only the image but also its semantic context, increasing interpretability and diagnostic reliability.

Furthermore, the system’s ability to maintain a transparent and immutable audit trail of all imaging data transactions addresses long-standing gaps in accountability. By recording access events on-chain, including timestamps, user identifiers, and data scopes, the architecture enforces traceability at a protocol level. Clinicians expressed trust in this visibility, recognizing its value for both internal governance and patient assurance.

The implications extend beyond clinical care. By validating that imaging data can be standardized, annotated, and shared within a framework that respects consent and privacy, the architecture creates a robust foundation for ethically aligned secondary uses. Annotated radiographs could be made available for federated learning without moving raw data across institutional boundaries, facilitating innovation in AI without breaching confidentiality. Researchers could reuse imaging data with appropriate permissions in place, streamlining ethical review processes and enabling broader participation in longitudinal studies.

From a deployment standpoint, the modular design of the system supports expansion to other imaging modalities such as CT or MRI. Its compatibility with off-chain encrypted storage and on-chain consent verification positions it well for integration with hospital information systems and national health infrastructures. Future work may explore the incorporation of ventilator telemetry, real-time physiological monitoring, and patient-reported outcomes to enrich the imaging context further.

In ethical and policy terms, the results offer evidence that security, privacy, and usability can be achieved simultaneously. The architecture operationalizes key principles of the FAIR data framework—making imaging data findable, accessible, interoperable, and reusable—without compromising on governance. As such, it may serve as a reference model for institutions aiming to modernize their data infrastructures in compliance with emerging norms around digital sovereignty and patient autonomy.

Ultimately, this work proposes a new model for medical imaging in digital health ecosystems: one where patients are empowered to manage access, clinicians can act swiftly with trust in data provenance, and researchers can ethically reuse high-value imaging datasets. The convergence of blockchain, FHIR, and digital identity technologies in this architecture presents a compelling vision for the future of imaging data management that is efficient, secure, and ethically aligned with the needs of modern healthcare.
